# Tregalizumab – A Monoclonal Antibody to Target Regulatory T Cells

**DOI:** 10.3389/fimmu.2016.00011

**Published:** 2016-01-25

**Authors:** Martin König, Faiza Rharbaoui, Silke Aigner, Benjamin Dälken, Jörg Schüttrumpf

**Affiliations:** ^1^Biotest AG, Dreieich, Germany

**Keywords:** monoclonal antibody, regulatory T cells, CD4 T cells, autoimmunity, rheumatoid arthritis

## Abstract

Regulatory T cells (Tregs) represent a subpopulation of CD4^+^ T cells, which are essential for the maintenance of immunological tolerance. The absence or dysfunction of Tregs can lead to autoimmunity and allergies. The restoration of functional Tregs and/or Treg cell numbers represents a novel and attractive approach for the treatment of autoimmune diseases, e.g., rheumatoid arthritis (RA). The CD4 cell surface receptor is a target for modulation of T cell function. Monoclonal antibodies (mAbs) against CD4 have previously been tested for the treatment of autoimmune diseases, including RA. Furthermore, in model systems, anti-CD4 antibodies are able to induce tolerance and mediate immunomodulatory effects through a variety of mechanisms. Despite the availability of innovative and effective therapies for RA, many patients still have persistently active disease or experience adverse events that can limit use. A growing body of evidence suggests that Treg modulation could offer a new therapeutic strategy in RA and other autoimmune disorders. Here, we describe tregalizumab (BT-061), which is a novel, non-depleting IgG1 mAb that binds to a unique epitope of CD4. Tregalizumab represents the first humanized anti-CD4 mAb that selectively induces Treg activation.

## Introduction

Regulatory T cells (Tregs) are an essential part of the immune system ensuring the maintenance of immunological tolerance and the prevention of autoimmunity (1). They are characterized by the expression of high levels of CD25 (IL2Ra), cytotoxic T lymphocyte-associated antigen 4 (CTLA-4), and glucocorticoid-induced tumor necrosis factor (TNF) receptor family α-related protein (GITR). Treg function and homeostasis relies on a stable expression of transcription factor Forkhead Box P3 (FoxP3) (2). Naturally occurring Tregs (nTregs) account for 5–10% of CD4^+^ T lymphocytes in the peripheral blood of healthy subjects (3). In the periphery, Tregs can convert from Foxp3^−^ T effector cells and are described as induced Tregs (iTregs). Several subpopulations of iTregs have been identified based on phenotypic and functional properties (4).

Functional imbalances of Tregs may contribute to the pathogenesis of rheumatoid arthritis (RA) and other autoimmune diseases (3). This has been described in rodents and is well documented in patients with RA (5, 6). Several studies indicate that Treg numbers are increased in the synovial fluid of RA patients, which is likely to be part of the response to inflammation. In RA, the increases of Treg numbers in synovial fluid were similar across diagnoses and disease durations (7). Subsequent studies have typically reported decreased or little change in the proportion of Tregs in the peripheral blood of RA patients compared with healthy controls (8).

Regulatory T cells from the synovial fluid of RA patients express an activated phenotype compared with those in peripheral blood from patients or healthy controls (9). FoxP3 mRNA, CTLA-4, OX-40, and GITR levels were also higher in the synovial fluid (9). Coculturing CD4^+^CD25^−^ cells with irradiated antigen-presenting cells (APCs) and anti-CD3 monoclonal antibodies (mAbs) demonstrated that Tregs from synovial fluid of RA patients were more suppressive than Tregs from peripheral blood (10). Subsequent analyses of Tregs derived from peripheral blood and synovial fluid from RA (9, 11) and juvenile idiopathic arthritis (JIA) patients (12) have indicated that Tregs retain their suppressive activity. Indeed, Treg function was markedly attenuated, which was reflected by an impaired ability to suppress T effector cell proliferation in patients with active RA compared with healthy controls (13).

The inflammatory milieu in RA synovium may render T cells more resistant to modulation by Tregs (14–16). TNF-α has been shown to abrogate the ability of Tregs to suppress T cell proliferation, although interleukin-6 (IL-6) had no effect (17). However, the effect of TNF-α on Tregs in mice and humans remains an area of discussion and controversy (18, 19).

## Therapies to Increase Treg Numbers and Function

The immunomodulatory influence of increased numbers of Tregs on immune responses in disease model systems has been extensively studied and represents an exciting immunotherapeutic strategy (20). Moreover, adoptive transfer of autologous or donor-derived Tregs as a cellular immunotherapy has been successfully studied in initial clinical trials in graft versus host disease (GVHD) (21, 22), underlining the therapeutic potential of Tregs. Other therapeutic strategies aimed at manipulating the existing Tregs (23, 24). A report on a high-throughput system identified several FDA-approved drugs that increase the number of Tregs with suppressive function (25).

In autoimmune disease, therapies with some approved biologics, including TNF-α inhibitors, have been shown to increase Treg numbers or function (26). For example, treatment with infliximab improved the ability of Tregs to inhibit cytokine production and increased the number of Tregs (27). Likewise, the human anti-TNF-α antibody adalimumab increased the percentage of FoxP3^+^ cells with restored regulatory function (28). By contrast, etanercept, a soluble TNF receptor, does not influence Treg cell number and function. It has been shown that TNFR2 expression is required for nTreg-mediated suppression and that TNF-α, in addition to stimulating Teff, is able to activate Tregs through TNFR2, which is preferentially expressed by Tregs. Furthermore, TNF-α is required as a critical factor in the activation of Tregs in tissue sites of inflammation (29). Anti-TNF-α therapy in human autoimmune diseases may therefore differentially affect the function of nTregs. Anti-TNF-α therapy is clinically effective in the management of autoimmune diseases (30); however, the mechanisms by which anti-TNF-α therapy exerts a clinical effect are currently not fully understood (29). The IL-6 inhibitor tocilizumab has been shown to be effective for the treatment of RA (31). Inhibition of IL-6 by tocilizumab can increase the number of Tregs, thereby restoring the Th17:Treg cell ratio in responding patients. Some approved biologics for the treatment of autoimmune disease therefore influence Treg numbers and function, contributing to their effectiveness.

## Biologics Targeting the T Cell Response

Current biologic RA therapies mainly target cytokines, including TNF-α (32), IL-1 (33, 34), and IL-6 (35). Recently, based on the apparent imbalance between Th17 and Treg activity (36), targeting IL-17 has been identified as another potential treatment intervention (32, 37), and anti IL-17 mAbs have been studied in RA but are not yet clinically available (38, 39).

Abatacept is a fusion protein of the CTLA-4 receptor and Fc domain of IgG1, which abrogates T cell costimulation, and thereby modulates T cell responses and interactions between T and B cells. It was approved in 2006 for the treatment of established (40–43) and early RA (44) and provides clinical “proof-of-concept” that targeting T cells is a rational treatment approach. The T cell receptor (TCR) interacts with CD3 to process signals resulting from an interaction with an antigen (38). Therefore, anti-CD3 therapy may be beneficial as an immunosuppressive agent. In mouse models of arthritis, anti-CD3 mAbs also reduce disease activity by induction of Tregs, leading to increased CD4^+^ and CD8^+^ Treg cells (45), and a transient downregulation of the TCR (46). Furthermore, anti-CD3 mAb therapy has induced remission in type I diabetes mellitus in non-obese diabetic (NOD) mice (47).

The clinical use of anti-CD3 mAbs has been limited by the induction of inflammatory cytokines and mitogenicity. Recent products have been developed to negate Fc receptor (FcR) functions, including complement-dependent cytotoxicity (CDC) and antibody-dependent cell-mediated cytotoxicity (ADCC). Teplizumab, an IgG1 anti-CD3 mAb with reduced FcR binding (48), has been assessed in type 1 diabetes and psoriatic arthritis. Treatment with teplizumab resulted in reduced C-peptide responses and a reduction in HbA1C levels in patients (49), but a further trial was halted due to an increased risk of adverse events, including lymphopenia. The Phase III PROTÉGÉ trial of teplizumab in type 1 diabetes failed to meet the primary end point, although there was evidence of benefit in certain subgroups (50). A dose-finding study using otelixizumab, a humanized CD3 mAb in type 1 diabetes, demonstrated reduced insulin requirements (51). However, a Phase III trial in type 1 diabetes (48) reported no significant benefit. Otelixizumab was also assessed in RA but development appears to be discontinued (52, 53).

These data indicate that anti-CD3 mAb treatment might be a feasible therapeutic approach; however, limited understanding of the potential mode of action of these mAbs in humans hinders interpretation of the studies to date (54).

The TCR coreceptor CD4 seems to be an attractive target for the modulation of T cell function. CD4 is widely expressed on T cells; therefore, anti-CD4 therapy might affect multiple subtypes, including potentially beneficial Tregs (55, 56). Current anti-CD4 mAbs act by coating the target CD4 molecule, making it inaccessible for ligands, downmodulating CD4, or depleting CD4^+^ target cells via induction of CDC or ADCC.

In mouse models of inflammatory arthritis, the anti-CD4 mAb YTS177 prevented or delayed onset of inflammatory disease (57), while in a rat kidney transplant model, the non-depleting mouse anti-rat CD4 mAb RIB5/2-induced long-term survival of the transplants. RIB5/2-induced tolerance was stable despite persistence of alloreactive T cells, suggesting a role of active tolerance-maintaining mechanisms. RIB 5/2-mediated tolerance can be adoptively transferred by Tregs isolated from the graft (58). The chimeric anti-CD4 mAb, priliximab (cM-T412) was not effective in RA patients, despite depleting peripheral CD4^+^ T cells at higher doses (59). However, a humanized non-depleting anti-CD4 mAb (OKTcdr4a) decreased CRP and resulted in transient clinical improvements in RA (60).

Keliximab, a primatized anti-CD4 mAb, demonstrated activity in RA patients in two randomized controlled trials and clinical responses correlated with CD4 T cell coating by keliximab (61). This suggests that non-depleting anti-CD4 mAbs could have therapeutic utility in RA. Clenoliximab, an IgG4 derivative of keliximab, modified to further reduce Fc-binding activity, demonstrated a reduced ability to downmodulate the CD4 receptor *in vitro*, but retained activity *in vivo* through dose-dependent CD4 “stripping” from the cell surface (62, 63). In a Phase II trial, clenoliximab induced American College of Rheumatology (ACR) criteria responses without CD4 depletion; however, this was not confirmed with long-term dosing (64). Another anti-CD4 mAb (4162W94) considered to be non-depleting resulted in sustained downmodulation of CD4 lymphocytes in an open-label pilot study (65). Although a placebo-controlled, repeat-cycle, follow-up trial demonstrated significant clinical activity in RA, unacceptable CD4 lymphopenia, and skin rashes lead to discontinuation of therapy (66).

Anti-CD4 antibodies with different pharmacodynamic (PD) properties have been investigated and shown to modulate T cell function in model systems and in clinical trials. However, treatment in clinical trials with the anti-CD4 mAbs investigated to date did not result in long-lasting clinical benefits.

## Tregalizumab

Tregalizumab represents a novel, humanized, anti-human CD4 IgG1 mAb, which binds to a unique epitope of CD4 in the IgG-like C2 type 1 domain (also known as D2) on the opposite side of the binding region for other known ligands, including other anti-CD4 mAbs, gp120, and MHC class II. This allows concurrent binding of a class II MHC molecule or a gp120 HIV-1 envelope protein (67). Tregalizumab is derived from a murine predecessor B-F5 by complementarity-determining region (CDR) grafting and subcloning.

Several effector functions of tregalizumab have been analyzed *in vitro*. Tregalizumab is unable to mediate induction of CDC, ADCC, or apoptosis in target cells (68). However, in contrast to other CD4 antibodies, the precursor antibody B-F5 and tregalizumab were both found to selectively activate Tregs (67, 69).

Regulatory T cells remain in an inactivated state and do not exhibit suppressive properties unless activated by appropriate signals via their TCR (70). *In vitro* incubation of Tregs with anti-CD3 antibodies is considered as an optimal stimulus to induce their suppressive activity, although anti-CD3 antibodies have no selectivity for Tregs and activate conventional T cells as well as Tregs.

**Figure 1:**
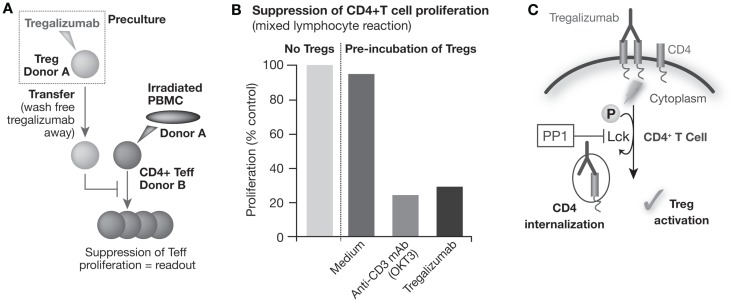
**Tregalizumab is able to activate the suppressive capacity of regulatory T cells**. **(A)** Tregs were isolated and pre-incubated with plate-bound tregalizumab, OKT-3 mAb, or medium and transferred to a mixed lymphocyte reaction using allogeneic, CD3-depleted, irradiated PBMCs to activate the proliferation of Teffs. **(B)** The proliferation of allogenic stimulated Teffs with tregalizumab pretreated Tregs is shown compared with the proliferation without Tregs. **(C)** Tregalizumab engages the TCR signaling pathway. Incubation of Tregs with tregalizumab evokes signaling events in Tregs that mimic a signal through the T cell receptor complex without the need for direct CD3 or TCR stimulation. T cell receptor complex signaling represents a crucial event in the activation of Tregs since blockade of the corresponding signaling pathway resulted in the loss of suppressive activity of Tregs. Tregalizumab delivers a comparable signal as usually conferred by TCR binding in Tregs, resulting in activation/phosphorylation of the T cell receptor downstream signaling molecule ZAP-70 (ζ-chain-associated protein). This event leads to signals that finally trigger internalization of the tregalizumab–CD4 complex. As a consequence, CD4 expression levels on CD4^+^ T cells are downmodulated by tregalizumab in the physiological setting. This downmodulation causes a transient decrease of CD4 molecules on the cell surface, which is followed by a recovery of expression levels over time (67, 69).

Using *in vitro* assays, it could be demonstrated that, in contrast to the other CD4 antibodies analyzed, tregalizumab can provide an activation signal selectively to Tregs (67, 69). Tregalizumab-treated Tregs strongly suppressed proliferation of CD4 and CD8 effector T cells following allogeneic or antigen-specific activation in mixed lymphocyte reactions (Figures 1A,B).

Cyclic AMP (cAMP) has been shown to be elevated in activated Tregs, which is then transmitted via gap junctions directly into responder T cells. The suppressive activity of nTregs can be abolished by cAMP antagonists as well as by gap junction inhibitors, which block the cell contact-dependent transfer of cAMP to responder T cells (71). *In vitro*, tregalizumab is able to activate Tregs as demonstrated by increased intracellular cAMP and Ca^2+^ levels, and by increased secretion of transforming growth factor beta (TGF-β) (67).

Tregalizumab recognizes the human CD4 molecule not only on Tregs but also on conventional T cells. Despite this, binding does not impair the proliferative capacity of these cells *per se*. In contrast to other anti-CD4 antibodies, tregalizumab does not inhibit the proliferation elicited by anti-CD3/anti-CD28 stimulation. Importantly, tregalizumab does not lead to the activation and proliferation of conventional T cells and does not induce the secretion of proinflammatory cytokines, in contrast to anti-CD3 mAbs. Indeed, tregalizumab reduced proliferation and cytokine secretion when isolated peripheral blood mononuclear cells (PBMCs) were stimulated with tetanus toxoid in a dose-dependent manner (68).

Incubation of Tregs with tregalizumab evokes signaling events in Tregs that mimic a signal through the TCR complex, without the need for direct CD3 or TCR stimulation (67, 69). TCR complex signaling represents a crucial event in the activation of Tregs since blockade of the corresponding signaling pathway, for example, via PP1, a specific src family kinase inhibitor, resulted in the loss of suppressive activity of Tregs (Figure 1C). Therefore, tregalizumab binding to CD4 triggers the induction of the signaling cascade in Tregs with the activation/phosphorylation of the TCR downstream signaling molecule ZAP-70 (ζ-chain-associated protein). *In vitro* studies have shown that several signaling molecules of the TCR pathway are engaged and become phosphorylated (67). When comparing commercially available anti-CD4 antibodies to tregalizumab, significant differences in signaling strength were observed. Although the phosphorylation signal on Lck was weakest with tregalizumab, it also mediated phosphorylation of LAT, SLP-76, PLC-γ, and MEK. However, tregalizumab did not induce phosphorylation of Itk, ERK, PKC, MAPK, or NF-κB, unlike anti-CD3 treatment or the other anti-CD4 antibodies tested (67). Therefore, only tregalizumab was able to induce suppressive properties of Tregs. Interestingly, to date, no signaling molecule has been found that was specifically activated in Tregs in comparison to CD4^+^ T effector cells, and both Tregs and T effector cells responded to tregalizumab with similar phosphorylation events.

Other antibodies targeting cell surface receptors trigger the internalization of the antibody–receptor complex, resulting in decreased receptor surface expression over time (72). The decrease of CD4 surface receptor expression mediated by tregalizumab is detectable *in vitro* after cross-linking using a secondary antibody. Neither isotype control antibodies nor the Fab fragment of tregalizumab were able to mediate CD4 downmodulation. *In vitro*, the maximal CD4 decrease was detected within a few hours and the magnitude of CD4 downmodulation seemed to be dose dependent, followed by a recovery over time. Interestingly, CD4 downmodulation occurs in both Treg and effector T cells without any observed differences in kinetics. CD4 downmodulation depends on tregalizumab-induced CD4 receptor signaling, which is triggered by the binding of tregalizumab to surface CD4. This specific signaling pathway is directly linked to the internalization event. PBMCs treated with tregalizumab in combination with the Lck-specific inhibitor PP1 led to a strong inhibition of CD4 receptor downmodulation. This indicates that the internalization of CD4 by tregalizumab depends on functional CD4 signaling and establishes a direct link between the activation of Tregs with CD4 downmodulation.

Downmodulation of CD4 expression levels has been reported in patients treated with tregalizumab (73), and this effect is of relevance as a marker for monitoring the activity of tregalizumab *in vivo*. Therefore, we hypothesized that the downmodulation of CD4 on the T cell surface could serve as a marker of tregalizumab PDs *in vivo*. This strategy was applied to establish a dose–response model of the CD4 modulation (73).

## Tregalizumab Clinical Development

The clinical development program of tregalizumab encompassed eight clinical studies: two in healthy subjects, two in psoriasis patients, and four in RA patients. Initial clinical data from Phase IIa dose-finding trials of tregalizumab in psoriasis (74) and RA (75) showed promising clinical effects. In a study of 55 patients with psoriasis who had failed to respond to systemic treatment, a single subcutaneous (SC) dose of tregalizumab (between 25 and 100 mg) induced psoriasis area and severity index (PASI) 50 responses in 19 patients, including 2 with PASI 75 as best observed responses. There was no evidence of increased cytokine levels or depletion of CD4^+^ T cells, and no increased risk of infections (74). Similarly, in a dose-finding study in patients with active RA who were disease-modifying antirheumatic drugs (DMARD) incomplete responders, tregalizumab monotherapy rapidly improved tender and swollen joint counts (75). At week 7, after 6 weeks of treatment, a maximum response of ACR 20/50/70 in the 50 mg SC dosing arm was achieved in 67, 33, and 17% of patients, respectively, compared to 14, 7, and 0% in the placebo arm. In some patients, improvements persisted beyond the dosing period.

**Figure 2:**
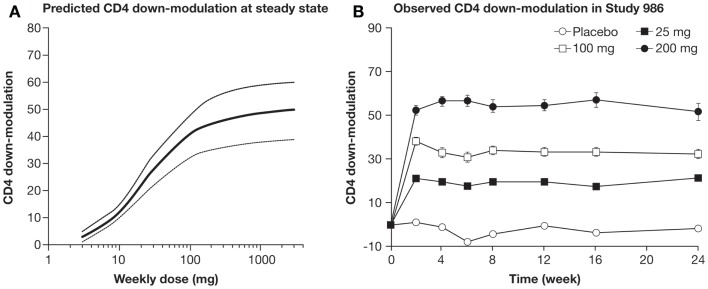
**(A)** PK/PD model was established to predict time course of CD4 downmodulation (% of baseline) after weekly dosing. **(B)** Mean CD4 downmodulation on CD4 T cells per dose group. CD4 modulation (±SEM) was measured by FACS analysis at weeks 0, 2, 4, 6, 8, 12, 16, and 24 just prior to the next dosing (77).

A large Phase IIb study with 321 patients with RA (TREAT 2b, T cell REgulating Arthritis Trial 2b) was initiated. TREAT 2b was a double-blind, randomized, placebo-controlled, Phase IIb trial with four treatment groups to evaluate the efficacy and safety of tregalizumab in methotrexate-inadequate responders. In the active dosing groups, tregalizumab was administered at SC doses of 25, 100, and 200 mg once weekly over 24 weeks in combination with methotrexate. Patients in the control arm received methotrexate only. In patients who responded to treatment, therapy was extended for a further 6 months (76). At week 12, none of the three dosing arms of tregalizumab showed a statistically significant improvement in ACR20 score (primary endpoint) when compared to placebo. Tregalizumab was generally well tolerated with usually mild-to-moderate adverse events balanced between placebo and treatment arms. Neither tuberculosis, opportunistic infections, major adverse cardiovascular events nor malignancies were reported during the study. No difference in infections between tregalizumab and placebo was observed. The results of tregalizumab in RA reported in the TREAT2b trial do not currently justify further clinical development in this indication due to lack of a responder population demonstrating sufficient efficacy. Nevertheless, the PD effects, including modulation of CD4 receptor expression that were observed in tregalizumab-treated patients, were as expected and consistent with predictions of established pharmacokinetic (PK)/PD modeling (73) (Figures 2A,B).

In summary, anti-CD4 antibodies demonstrate different PD properties and several mechanisms have been investigated in clinical trials to influence T cell-driven diseases. In these studies, treatment with anti-CD4 mAbs did not result in significant clinical benefit. Tregalizumab represents a mAb with a novel and unique mode of action and signs of efficacy have been observed in Phase II trials in psoriasis and RA. However, statistically significant efficacy could not be confirmed in the larger trial in patients with RA. Antibody activity was demonstrated in all trials by measuring CD4 modulation as a PD marker and a PK/PD model system was also established to predict the dose–response of CD4 modulation. In parallel, the safety profile observed in earlier studies has been confirmed in the larger trial. More mechanistic and clinical data are needed to better understand applicability of this novel therapeutic approach; however, the unique mechanism of tregalizumab remains attractive and should be explored in further diseases in which insufficient Treg activity is postulated as a pathophysiological mechanism.

## Author Contributions

MK: wrote the manuscript, designed the work, analyzed and interpreted the data, and approved the manuscript. FR, SA, and BD: designed the work, analyzed and interpreted the data, revised and approved the manuscript. BD was accountable for all aspects of the work. JS analyzed and interpreted the data, revised and approved the manuscript, and was accountable for all aspects of the work.

## Conflict of Interest Statement

Martin König, Faiza Rharbaoui, Silke Aigner, Benjamin Dälken, and Jörg Schüttrumpf are employees of Biotest AG, Dreieich, Germany.
